# Monte Carlo-based quantitative pinhole SPECT reconstruction using a ray-tracing back-projector

**DOI:** 10.1186/s40658-017-0198-z

**Published:** 2017-12-15

**Authors:** Mikael Peterson, Johan Gustafsson, Michael Ljungberg

**Affiliations:** Department of Medical Radiation Physics, Lund University, SE-221 85 Lund, Sweden

**Keywords:** Monte Carlo simulations, SPECT reconstruction, Monte Carlo-based reconstruction, Pinhole SPECT reconstruction, Dual-matrix reconstruction, Myocardial perfusion SPECT imaging

## Abstract

**Background:**

Monte Carlo simulations provide accurate models of nuclear medicine imaging systems as they can properly account for the full physics of photon transport. The accuracy of the model included in the maximum-likelihood–expectation-maximization (ML-EM) reconstruction limits the overall accuracy of the reconstruction results. In this paper, we present a Monte Carlo-based ML-EM reconstruction method for pinhole single-photon emission computed tomography (SPECT) that has been incorporated into the SIMIND Monte Carlo program. The Monte Carlo-based model, which accounts for all of the physical and geometrical characteristics of the camera system, is used in the forward-projection step of the reconstruction, while a simpler model based on ray-tracing is used for back-projection. The aim of this work was to investigate the quantitative accuracy of this combination of forward- and back-projectors in the clinical pinhole camera GE Discovery NM 530c.

**Results:**

The total activity was estimated in ^99m^Tc-filled spheres with volumes between 0.5 and 16 mL. The total sphere activity was generally overestimated but remained within 10% of the reference activity defined by the phantom preparation. The recovered activity converged towards the reference activity as the number of iterations increased. Furthermore, the recovery of the activity concentrations within the physical boundaries of the spheres increased with increasing sphere volume. Additionally, the Monte Carlo-based reconstruction enabled recovery of the true activity concentration in the myocardium of a cardiac phantom mounted in a torso phantom regardless of whether the torso was empty or water-filled. A qualitative comparison to data reconstructed using the clinical reconstruction algorithm showed that the two methods performed similarly, although the images reconstructed using the clinical software were more uniform due to the incorporation of noise regularization and post-filtration in that reconstruction technique.

**Conclusions:**

We developed a Monte Carlo-based reconstruction method for pinhole SPECT and evaluated it using phantom measurements. The combination of a Monte Carlo-based forward-projector and a simplified analytical ray-tracing back-projector produced quantitative images of acceptable image quality. No explicit calibration is necessary in this method since the forward-projector model maintains a relationship between the number of counts and activity.

## Background

An attractive, but seldom clinically used, aspect of single-photon emission computed tomography (SPECT) is that it can be utilized for activity quantification in vivo, with applications in, e.g. radionuclide therapy dosimetry [1, 2] and myocardial perfusion studies [3]. The accuracy of SPECT-based activity quantification depends on the accuracy with which radiation transport in the patient and the detector are modelled in the tomographic reconstruction algorithm. The level of accuracy achievable varies with the complexity of the emission photon spectrum of the radionuclide used. Typical photon energies in SPECT imaging range from 50 to 400 keV. Within this energy range, there is a considerable probability that an emitted photon will interact within the patient before reaching the detector, which is difficult to model accurately. Furthermore, the finite hole diameter of the collimator causes substantial degradation of the spatial resolution of the image. High-energy photons, which have non-negligible probabilities of penetrating the collimator septa, further degrade the image quality. For imaging using radionuclides that emit photons of multiple energies, high-energy photons can be down-scattered into lower energy windows. Hence, all of these phenomena must be considered to achieve reliable activity quantification. The accuracy is further limited by image noise, uncertainties associated with system calibration, and patient motion. The absolute quantitative accuracy of gamma camera SPECT quantification has typically been reported to be within 20% for a range of radionuclides [2, 4].

The collimators typically employed in clinical SPECT are parallel-hole collimators, which is also the case for clinical quantitative SPECT. Pinhole collimators also have great potential. Historically, pinhole imaging has been focused on either superficial small organs (e.g. the thyroid gland) or pre-clinical imaging of small animals (e.g. rats and mice). However, pinhole collimation offers several advantages over parallel-hole collimation, such as increased system sensitivity and superior spatial resolution, but at the cost of a smaller field of view (FOV) [5–7]. During the last two decades, renewed interest in pinhole imaging techniques has developed in the nuclear medicine community, as well as in their clinical applications, following extensive hardware developments driven primarily by preclinical research [8, 9]. These developments include new detectors and detector materials [9], advanced pinhole and multi-pinhole geometries [10, 11], and the implementation of iterative reconstruction methods [12, 13]. In the clinical realm, recent pinhole camera-system research has focused on myocardial perfusion imaging. In the commercially available system GE Discovery NM 530c (NM 530c) [14], a pinhole collimator and cadmium zinc telluride (CZT) semiconductor detector are employed. These compact detectors have made it possible to mount multiple detector-pinhole units in a static geometry to acquire projections simultaneously with an angular coverage sufficient for tomographic reconstruction. The NM 530c camera has a sensitivity three times higher than those of conventional dual-headed SPECT cameras equipped with parallel-hole collimators and NaI crystals [14]. However, the small FOV, a spherical volume 19 cm in diameter, limits its application to specific organs of interest. Since all of the projections are acquired simultaneously, NM 530c enables dynamic myocardial blood flow and perfusion studies to be conducted [15, 16], which are otherwise only feasible using positron emission tomography (PET) [17]. An accurate quantitative reconstruction method is a prerequisite for model-based kinetic analysis.

Quantitative SPECT requires attenuation and scatter correction. Typically, SPECT attenuation correction is performed using a co-registered attenuation map based on computed tomography (CT) images. For scatter correction, several correction methods exist [18]. Approximate window-based methods are often employed for gamma cameras. For CZT detectors, the presence of un-scattered photons with energies below the primary energy window will introduce bias when utilizing window-based methods [19]. This bias has been addressed by developing new correction methods for CZT-based systems, including modified window-based methods [20] and iterative deconvolution approaches [21].

The most commonly used iterative reconstruction algorithms in nuclear medicine applications are the maximum-likelihood–expectation-maximization (ML-EM) [22] algorithm and the closely related ordered-subsets–expectation-maximization (OS-EM) algorithm [23]. These methods have been extensively described in the literature, see for example [24]. Starting from an initial estimate, the activity distribution is iteratively updated, and each iteration involves a series of operations that can be summarized as

1 $$ {f}_j^{\mathrm{new}}=\frac{f_j^{\mathrm{old}}}{\Sigma_i{a}_{ij}}{\Sigma}_i{a}_{ij}\frac{p_i}{\Sigma_k{a}_{ik}{f}_k^{\mathrm{old}}}, $$

where ${f}_j^{\mathrm{old}}$ is the current estimated value in voxel *j*, *p*_*i*_ is the measured value in projection pixel *i*, ${f}_j^{\mathrm{new}}$ is the updated value of voxel *j*. Since the method is based on comparing calculated projections with measured projections, an accurate model of the image process, i.e. a SPECT system, is essential. This model is described in Eq. 1 as a system matrix with elements *a*_*ij*_, where each element represents the probability that a decay in voxel *j* will result in a detected photon in projection pixel *i*. Any mismatch between the projection formation process as reflected by the system matrix and the true projection formation process by the actual SPECT system will propagate to the reconstructed image and result in bias. Hence, for the reconstructed image to reflect the true activity distribution and for the values in that image to be useful for quantitative purposes, accurate modelling of the system and radiation transport from decay to detection is necessary.

In terms of radiation transport modelling, one of the most trusted strategies is to use the Monte Carlo (MC) method, since it enables inclusion of the details of the measurement geometry and physics governing photon interactions. The potential benefits of MC-based SPECT reconstruction was discussed already in the 1980s by Floyd et al. [25] but the procedure was limited to reconstructing a single slice. Even though the computational burden of MC simulations still poses a challenge for full Monte Carlo-based reconstruction (MCR), the continuous improvements in computer hardware and the possibility of parallel processing have made this method increasingly interesting for practical use.

An MC simulation can be included in an ML-EM algorithm in a variety of ways. For example, individual components, such as the scatter contribution, can be modelled using MC methods while deriving the remaining components of the projection model by other means [26–29]. Alternatively, the forward-projection can be fully MC-based, possibly with some approximations to increase the computational efficiency [30, 31], or an MC-based pre-calculated system matrix can be utilized in both the forward projection and the back projection [32–35].

One MC program that is optimized for the simulation of gamma cameras is SIMIND [36]. In the SIMIND code, several variance reduction techniques are implemented to reduce the simulation time, as described in [37]. Recently, a model of CZT photon detection was incorporated into the SIMIND code [38]. In short, the CZT charge collection following a photon interaction is modelled using the Hecht equation [39] and lateral charge diffuses according to a distance-dependent Gaussian distribution sampling. The CZT model in the SIMIND code has been validated for the NM 530c system based on the known geometry of the system [38]. Furthermore, the SIMIND pinhole modelling has been verified using analytical expressions and published data [40].

While it is theoretically possible to pre-compute the system matrix for a given (patient-specific) geometry, the storage requirements increase dramatically with the image and projection matrix sizes [33]. The typical mode of operation is instead to compute forward- and back-projections during the reconstruction process without reference to a pre-computed system matrix. A problem that is evident in MCR, due to the nature of the simulation procedure, is then that the MC program is only directly applicable as a forward-projector, and its use as a back-projector would be computationally cumbersome. Hence, the back-projector often must be derived in a more simplistic fashion.

The use of different models in the forward- and back-projectors is referred to as dual-matrix reconstruction [41]. It has been demonstrated that approximations can be introduced into a back-projector as long as the model used in the forward-projection is accurate. For example, including attenuation modelling in the back-projector often has only a small effect on the reconstruction results, while it is generally beneficial to model the spatial resolution in the back-projector [41, 42]. Mathematically, for dual-matrix reconstruction, the counterpart of Eq. 1 becomes

2 $$ {f}_j^{\mathrm{new}}=\frac{f_j^{\mathrm{old}}}{\Sigma_i{b}_{ij}}{\Sigma}_i{b}_{ij}\frac{p_i}{\Sigma_k{a}_{ik}{f}_k^{\mathrm{old}}}, $$

where *b*_*ij*_ denotes the elements of the system matrix used in the back-projection.

In this study, we developed and extended the functionality of SIMIND to include tomographic pinhole reconstruction. Our aims were to take advantage of the accurate forward-projection modelling provided by a full MC simulation with the SIMIND program and to combine it with an approximate back-projector, thereby creating an integrated program for MC-based pinhole SPECT reconstruction. We then evaluated the reconstruction method with respect to its quantitative accuracy using phantom measurements. Qualitative comparison with the clinical reconstruction program of the camera was also performed.

## Methods

### GE discovery NM 530c

The GE Discovery NM 530c [14] consists of 19 detector-pinhole units, and each detector has 32 × 32 individual detector elements in a 78.7 mm × 78.7 mm area. The detectors are arranged in five detector triplets alternating with four single detectors, and the whole detector package is curved around the torso of the patient, providing angular coverage of 180° with a 19-cm-diameter spherical FOV. The lateral detectors in each triplet, of which there are 10 in total, are tilted relative to the pinhole axis.

Clinical image reconstruction is performed using an iterative Bayesian (‘one-step-late’) algorithm [42, 43]. The reconstructed images have a matrix size of 70 × 70 × 50 with 4 mm × 4 mm × 4 mm voxels. It is possible to include an attenuation correction in clinical ML-EM reconstruction, but doing so requires a CT image to be acquired on a separate system, and consequently, the attenuation coefficient map must be co-registered with the SPECT volume prior to reconstruction. A SPECT/CT version of NM 530c, called NM 570c, is commercially available. Without accurate attenuation and scatter correction and a calibration coefficient, reconstructed data cannot be used for activity quantification. In this study, neither attenuation nor scatter correction was included in the NM 530c reconstruction.

### SIMIND ML-EM

The pinhole reconstruction method described in this work was fully incorporated into the SIMIND MC code and operates as a stand-alone program. Thus, the reconstruction program can take advantage of all future improvements and updates in the main SIMIND code.

Three separate files are used to define the system and SPECT acquisition. The first two are parts of the general SIMIND framework for pinhole SPECT simulation and describe (a) the source and detector characteristics and (b) the detector and pinhole positions and orientations. A third file, stored in interfile 3.0 format, provides study-specific information, such as the acquisition time and energy window settings.

The reconstruction starts with loading the measured projection data and an optional co-registered CT. A three-dimensional source volume with a uniform positive value is used as a first estimate. The SIMIND program then simulates projections scaled with the proper acquisition time of the measured projections. In this particular work, the projections were simulated for the NM 530c by including all of the physical effects modelled using SIMIND, such as non-homogeneous photon attenuation, scatter in the object, scatter and penetration in the collimator, interactions in the CZT material, and the subsequent charge transport (including electron- and hole-trapping and charge diffusion).

The description of the system design and detector geometry specifications was provided by GE Healthcare, Haifa, Israel.

#### Back-projector

As opposed to the MC-based forward-projector, the back-projector model includes several approximations. These are that (a) no attenuation or scatter occurs in the object, (b) the pinhole aperture opening is infinitely small without penetration effects (i.e. perfect collimation), and (c) the efficiency is independent of the incident angle. An analytical expression for the pinhole point spread function (PSF), including penetration through the pinhole edges, has been described elsewhere [43] but was not considered in this study due to its complexity in relation to the NM 530c geometry. Instead, we used a more approximate ray-tracing algorithm to derive the individual elements of *b*_*ij*_.

The probabilities *b*_*ij*_ are derived on a voxel-by-voxel basis for an *N* × *N* × *N* source matrix with voxel side lengths *m*. A focal line originating at point **P** will intercept the plane of an individual detector at point **D** by passing through the infinitesimally small pinhole. The line from the source volume origin through the pinhole centre is referred to as the pinhole axis, and the direction of the pinhole axis is specified by a polar angle *θ* and an azimuthal angle *φ*. The coordinates of **D** are calculated from those of **P** using the detector-to-pinhole distance **H** and origin-to-pinhole distance **R**. For this purpose, the coordinate system is rotated so that the *z* ^′^-axis becomes parallel to the pinhole axis. The new coordinates **P** ^′^ after rotation of the reference system about the *x*-axis and *y*-axis are given by

3 $$ \left[\begin{array}{c}{x}^{\hbox{'}}\\ {}{y}^{\hbox{'}}\\ {}{z}^{\hbox{'}}\end{array}\right]=\left[\begin{array}{ccc}\cos \varphi & \sin \varphi \sin \theta & \sin \varphi \cos \theta \\ {}0& \cos \theta & -\sin \theta \\ {}-\sin \varphi & \cos \varphi \sin \theta & \cos \varphi \cos \theta \end{array}\right]\left[\begin{array}{c}x\\ {}y\\ {}z\end{array}\right], $$

The coordinates of **D** are calculated by mirroring and then scaling *x* ^′^ and *y* ^′^ by a magnification factor *M*:

4 $$ {x}_{\mathrm{D}}=-{Mx}^{\hbox{'}}, $$

5 $$ {y}_{\mathrm{D}}=-{My}^{\hbox{'}}, $$

6 $$ M=\frac{H}{R-{z}^{\hbox{'}}} $$

Due to the finite size of the source voxels, photons originating from a single voxel can contribute to several projection bins. This behaviour is modelled by adapting a scalable grid centred on the intercept point, and the width of the grid *G*_D_ is calculated by scaling *m* by *M*:

7 $$ {G}_{\mathrm{D}}= mM. $$

**Fig. 1:**
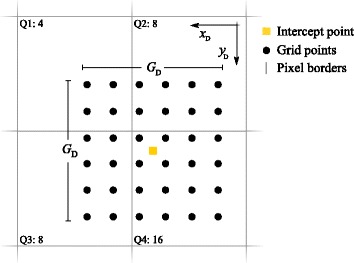
Schematic representation of the *N*_grid_ × *N*_grid_ grid points (black dots) centred about the detector plane intercept point (yellow square). The grid is, in this case, distributed over four pixels (whose borders are represented by the grey lines), and the numbers of grid points in the respective pixels are 4, 8, 8, and 16 for quadrants 1, 2, 3, and 4 (Q1, Q2, Q3, and Q4), respectively. The signal contribution to a voxel from a specific pixel is the product of the pixel value and a normalization factor, that is, the number of grid points in the pixel divided by the total number of grid points in all of the detectors for the current voxel

The grid procedure is illustrated in Fig. 1, where the yellow point represents the detector intercept, with *N*_grid_ × *N*_grid_ black grid points distributed over the *G*_D_ × *G*_D_ area.

The number of points within the *G*_D_ × *G*_D_ grid is calculated using the heuristic equation,

8 $$ {N}_{\mathrm{grid}}=\left\lfloor 2+\frac{60}{1+{\left(R-{z}^{\hbox{'}}\right)}^{0.8}}+0.5\right\rfloor, $$

where ⌊⋅⌋ denotes truncation to the nearest lower integer. The total number of grid points (*N*_grid_ × *N*_grid_) should be inversely proportional to the squared voxel-to-pinhole distance (*R* − *z* ^′^)^2^ to model the distance-dependent sensitivity of the pinhole. However, accurate modelling of this sensitivity would require a large number of grid points for voxels located close to the pinhole, which would result in long calculation times. Instead, Eq. 8 was adopted to reduce the back-projection computation time. The constant 2 in Eq. 8 is included to ensure that the total number of grid points is greater or equal to 2^2^ for all voxel positions, whereas the maximum number of grid points is never greater than 62^2^. The constant 0.5 in Eq. 8 assures rounding to nearest integer. It should be noted that Eq. 8 does not account for the decreased sensitivity for oblique angles.

The probability *b*_*ij*_ is finally obtained by normalizing the number of sample points in a pixel *n*_*ij*_ to the total number of sample points on all projections for a given *j*, i.e. ${\sum}_{k=0}^{K-1}{N}_{\mathrm{grid},j,k}^2$ where the sum goes over the all *K* projections

9 $$ {b}_{ij}=\frac{n_{ij}}{\sum_{k=0}^{K-1}{N}_{\mathrm{grid},j,k}^2}. $$

#### Source estimate update

The new source estimate is the product of the current source estimate and correction factors calculated from the back-projected ratios, according to Eq. 2, and is stored as two datasets. One dataset is stored as floating-point single-precision values, with each voxel value equalling the activity in MBq in the volume represented by that voxel, while the other dataset is stored as integers, where the sum of the integer values is the predefined number of histories for the MC simulation. The SIMIND program is then restarted using the latter of the two source files as the input. In this way, the SIMIND program continuously maintains an accurate relation between the number of histories generating counts in the projection images and the underlying activity in a particular voxel in the source volume. Hence, no additional calibration factor is needed to obtain absolute values of the activity in each reconstructed source voxel. The procedure is repeated until the predefined number of iterations has been conducted.

### Simulations and measurements

For MCR, measured projections were reconstructed into a 70 × 70 × 70 matrix with 4-mm cubic voxels. This voxel size is identical to that in the reconstructions produced by NM 530c systems (70 × 70 × 50 and 4 mm). For qualitative comparison, the measured projections were also reconstructed using the clinical reconstruction algorithm, which involves 40 iterations of a regularized (*α* = 0.51 and *β* = 0.3) one-step-late Green ML-EM algorithm [44] and post-filtered using a Butterworth filter (cut-off: 0.37, order: 7).

#### Simulated data

To ensure consistency in the reconstruction algorithm as such, SPECT projections were simulated using SIMIND and then reconstructed using SIMIND MCR, meaning that the forward-projector utilized in the reconstruction was identical to the projector employed to create the projections. The purpose of reconstructing simulated data is to isolate any causes of inaccuracy to either the forward projector or back projector. If the estimated activity from a simulated reconstructed sphere is close to the reference value it means that the back projector is properly normalized. On the other hand, if the relative difference is off by a factor similar to that of measured spheres, this would indicate that the back-projector is not correctly normalized since the forward projector in this case can be considered “perfect”. In this study, a 9-mL sphere was centred at seven different positions, namely, at the reconstruction volume centre and at six positions with 2 cm positive or negative translations along the *x*-, *y*-, and *z*-axes. The activity was defined as 1 MBq, and simulated projections were calculated for each position with the sphere both in a non-attenuating medium, subsequently called ‘air’, and in a cylindrical water phantom (21.4 cm in the axial direction, 11.0 cm radius) with a density of 1 g/cm^3^. Sufficiently many histories were simulated to attain essentially noise-free projections. No Poisson distributed noise was added to the projections.

Each projection set was reconstructed 10 times to determine the variance in the recovered total activity due to the finite number of photon histories in the SIMIND forward-projection step during MCR. The reconstructed sphere activity was determined using a spherical volume of interest (VOI) with twice the diameter of the sphere used in the simulations, and from this VOI, the relative differences between the estimated activities and those defined in the simulations were calculated.

#### Point source measurements

The correct placement of the activity in the reconstructed images was investigated using a point-like source, in the form of a 600-μm-diameter ion resin bead soaked in ^99m^Tc-pertechnetate and placed on the tip of a plastic rod. The rod was in turn mounted on a motorized 3D translational table, and projections of 125 source positions, defined as a 5 × 5 × 5 cubic grid with 20 mm spaces between the points, were obtained.

The acquired projections were reconstructed using 5, 10, 15, and 20 iterations, and the source volume was filtered using a 3D Gaussian filter with a standard deviation equal to the side length of a voxel in a 7 × 7 × 7 kernel. To investigate the spatial integrity of the reconstruction, we (a) determined the voxel coordinates for the maximal value in each reconstructed volume for each source position, (b) calculated the mean coordinate position of a plane along the coordinate system axis orthogonal to that plane as the mean of the 25 voxel coordinates (repeated for all planes orthogonal to one of the coordinate system axes), and (c) determined the distance between adjacent planes.

#### Sphere phantom

The quantitative accuracy of the MCR was investigated using six spheres with volumes ranging from 0.5 to 16 mL (NEMA standard) that were filled with ^99m^Tc and each of which had the same activity concentration. The spheres were mounted in an elliptical torso phantom and measured with and without water present in the torso, the activity concentrations being 4.1 and 3.8 MBq/mL, respectively. The sphere-mounting disk was designed so that the spheres could be mounted in the torso phantom using the original cardiac mounting bracket. The angulation of the mounting disk was such that a plane through the centre of the spheres was the same as the cardiac short-axis plane.

The MC simulations of photon interactions in SIMIND rely on a combination of tabulated material and energy-specific mass-attenuation coefficients and a density distribution. A CT study of the phantom was performed, and the results were co-registered to the clinically reconstructed SPECT images using the GE Xeleris™ workstation. The results were then exported by GE Xeleris™ as attenuation images and rescaled to density distributions.

In this study, we defined the activity recovery coefficient (ARC) as the ratio between the estimated and reference sphere activities. The ARC indicates how well the MCR recovers the activity within a sphere and was determined for each sphere using

10 $$ \mathrm{ARC}=\frac{A_E}{A_R}, $$

where *A*_*E*_ and *A*_*R*_ are the estimated and reference activities, respectively. Two sets of spherical VOIs were used. In one set, the VOI diameter was equal to the physical diameter of the sphere, and in the second set, an additional 8 mm (the size of two voxels) was added to the diameter of each VOI to collect counts otherwise lost due to the spatial resolution. The coefficients obtained from the second type of VOIs are called total activity recovery coefficients (TARCs). Each of the two types of VOIs was generated by manually defining the sphere centre from the images and then producing a spherical VOI mask based on the sphere radius.

#### Cardiac phantom

Measured projections of a myocardial phantom (Data Spectrum Corp, Hillsborough, NC, USA) were used to enable quantitative MCR evaluation and to compare the MCR to the NM 530c reconstruction. The 120-mL myocardium cavity was filled with a ^99m^Tc solution and imaged twice. For the first acquisition, the torso was filled with water, and the activity in the myocardium at the start of acquisition was 25.2 MBq. For the second acquisition, there was no water in the torso cavity except in the heart insert, and the activity at the start of acquisition was 21.1 MBq. The ventricle cavity was water-filled during both measurements. The thickness of the myocardium compartment was 10.3 mm, and the width of the ventricle compartment was 38.0 mm.

Reconstructed images (40 iterations) were re-oriented and overlaid on the CT image to enable visual confirmation of the spatial registration between the two modalities. Line profiles calculated as the average of seven rows over the myocardium was plotted for different iterations.

The MCR reconstruction was compared to the clinical NM 530c reconstruction of these measurements by plotting a line profile (an average of seven rows) in a long-axis slice. Prior to this comparison, the MCR images were filtered using a Butterworth low-pass filter (cut-off frequency 0.5 cm^−1^, order 2).

## Results

### Simulated data

Table 1 shows the relative differences between the estimated activities and those determined based on the reconstruction of SIMIND-simulated projections. The standard deviation of the estimated activity due to the finite number of histories in the forward-projection was deemed negligible (< 2 kBq) and is not shown in the table.

**Table 1:** Relative differences between estimated and simulated activities in a 9-mL sphere. The projections were simulated using a separate SIMIND simulation and then reconstructed using MCR (20 iterations)

Offset (cm)			Air	Water
*x*	*y*	*z*	Relative difference (%)	Relative difference (%)
0	0	0	1.8	0.7
−2	0	0	2.0	0.8
2	0	0	2.1	0.8
0	−2	0	1.8	0.5
0	2	0	1.7	0.7
0	0	−2	2.1	0.8
0	0	2	1.7	0.4

### Point source measurements

**Fig. 2:**
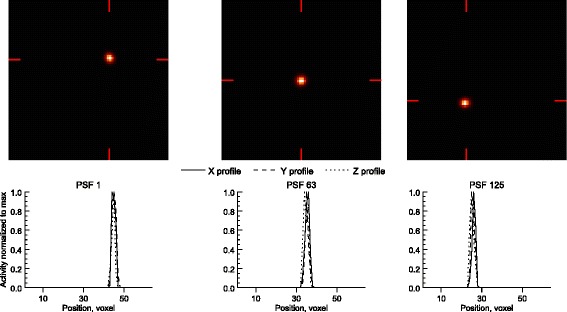
Maximum intensity projections of reconstructed point sources, in the *x*-*y*-plane, for the first, middle, and final source positions in the measurement grid. Line profiles are shown for the *x*- (solid), *y*-(dashed), and *z*- (dotted) directions with the positions of the former two marked by the red indicators. Each profile was normalized to have a maximum value of 1. The reconstructed point sources are symmetric in all directions, and no major artefacts are visible

Table 2 lists the calculated distances between pairs of adjacent planes, together with the standard deviations of the differences. The calculated distances never differ by more than 3 mm from the actual 20 mm distance for any plane combination, and the standard deviation is always less than the side length of one voxel (4 mm). For visual clarity, only the results for 20 iterations are shown, but there were no major differences in spatial integrity for different numbers of iterations. Figure 2 depicts the reconstructed point sources (maximum intensity projections) for the first, central, and last positions in the cubic measurement grid together with the corresponding *x*-, *y*-, and *z*-profiles. The reconstructed point sources are symmetrical in all directions and the reconstructed image is free of major artefacts.

**Table 2:** Calculated distance between point source planes (in millimeters)

Planes	1 and 2	2 and 3	3 and 4	4 and 5
*X-Y*	19.8 ± 0.3	19.5 ± 0.4	18.2 ± 0.5	18.7 ± 0.4
*X-Z*	20.0 ± 0.0	19.7 ± 0.2	19.7 ± 0.4	17.8 ± 0.5
*Y-Z*	20.0 ± 0.2	20.0 ± 0.2	19.8 ± 0.2	20.0 ± 0.0

### Sphere phantom

**Fig. 3:**
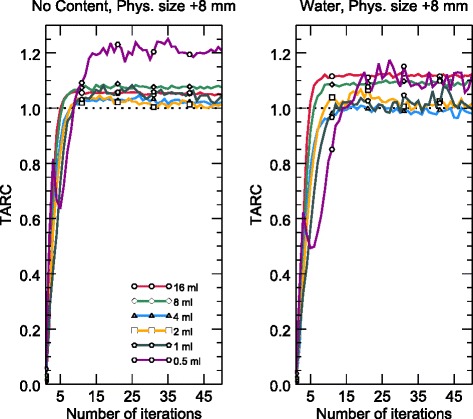
TARCs for spherical VOIs with diameters equal to the physical diameter plus 8 mm (the length of two voxel sides). The results are shown for iterations 1–50 for a set of spheres in the empty (left) and water-filled (right) torsos. Only every fifth data point is marked for clarity. The TARCs rise initially, up to 10–15 iterations and remained constant up to 100 iterations, but only the initial 50 iterations are shown here

**Fig. 4:**
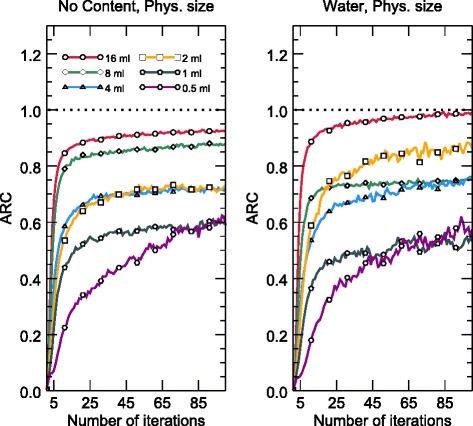
ARCs for spherical VOIs with diameters equal to the sphere diameter. The results are shown for iterations 1–100 for spheres in the empty (left) and water-filled (right) torsos. Only every 10th data point is marked for better clarity. The ARCs increase rapidly initially and continue to increase up to the user-selected endpoint of 100 iterations

**Fig. 5:**
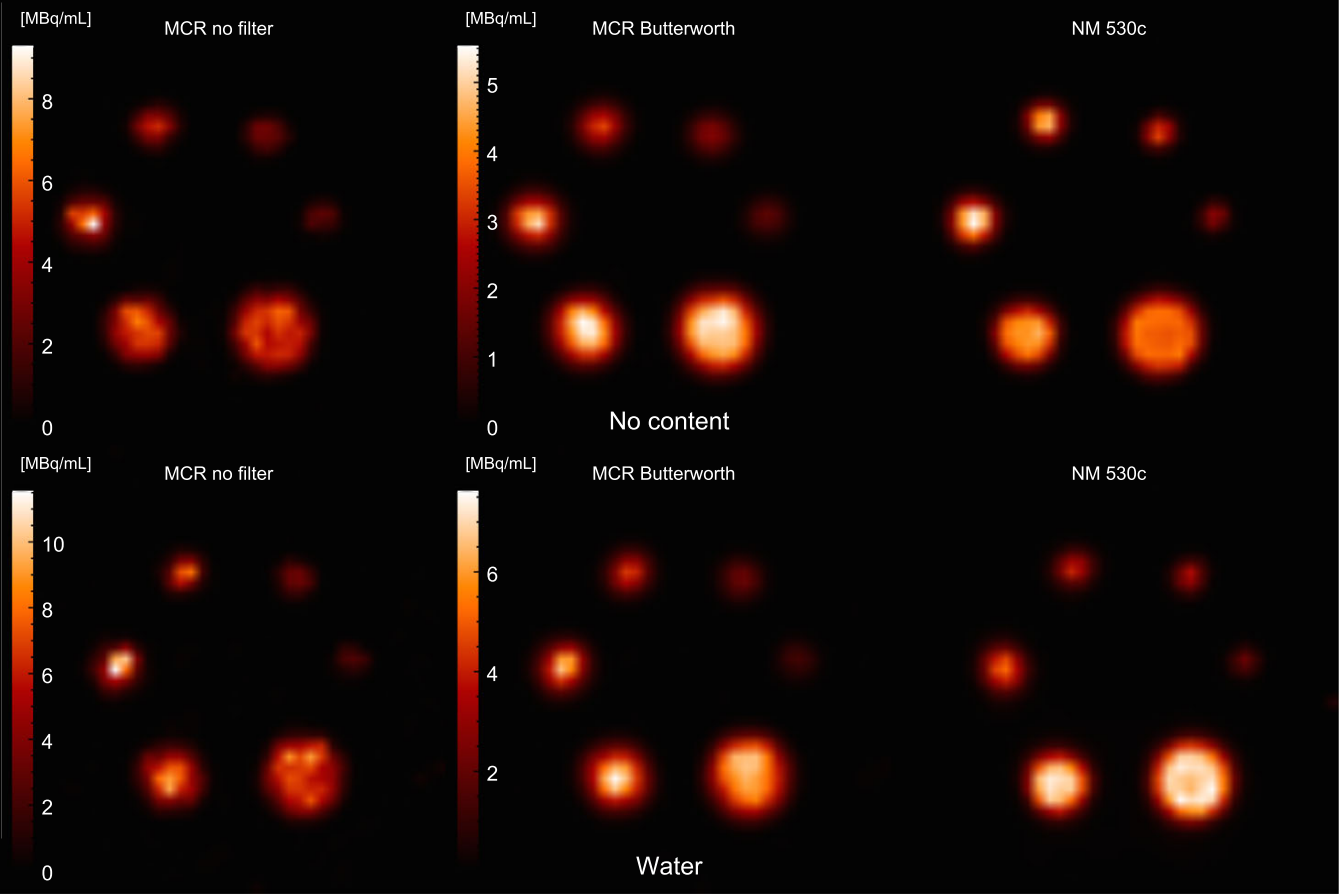
Maximum-intensity projection images for spheres in empty (top) and water-filled (bottom) torsos reconstructed using 40 iterations of MCR (left and middle) and NM 530c reconstruction (right). The activity concentrations were 3.8 and 4.1 MBq/mL at the start of the measurement for the spheres in the empty and water-filled torsos, respectively. The sphere volumes were 16, 8, 4, 2, 1, and 0.5 mL, moving clockwise from the lower-right sphere in each image. MCR images are shown with and without Butterworth post-filtration

The TARCs and ARCs are plotted in Figs. 3 and 4, respectively, as functions of the number of iterations for the six sphere sizes. The left and right plots in each figure present the measurements obtained using empty and water-filled torsos, respectively. The dotted lines indicate perfect recovery. Note that Fig. 3 shows data for iterations 1–50 to highlight the rapid TARC increases between 1 and 10 iterations, but above 10 iterations, the TARCs remain stable up to at least 100 iterations, which was the selected end point. Figure 5 presents maximum-intensity projections of the reconstructed spheres for empty and water-filled torsos obtained via MCR and NM 530c reconstruction. MCR reconstructed images are shown both as unfiltered and with Butterworth filter (cut-off frequency 0.5 cm^−1^, order 2). The number of iterations is 40 in all six cases.

### Cardiac phantom

**Fig. 6:**
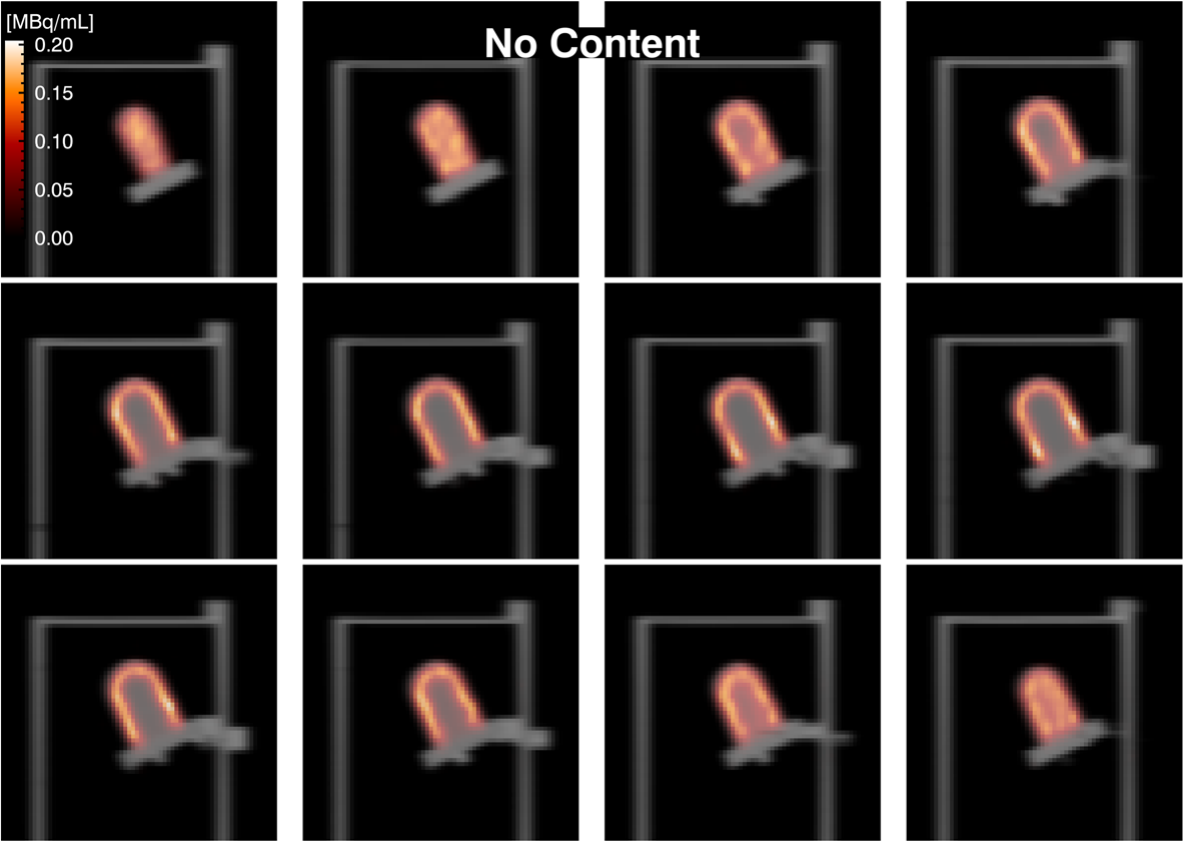
Twelve consecutive SPECT slices (50 iterations) overlaid on the corresponding CT slices for a cardiac insert in an empty torso. The ventricle cavity was filled with water. The MCR and CT results are well registered with good contrast between the myocardium and ventricle

**Fig. 7:**
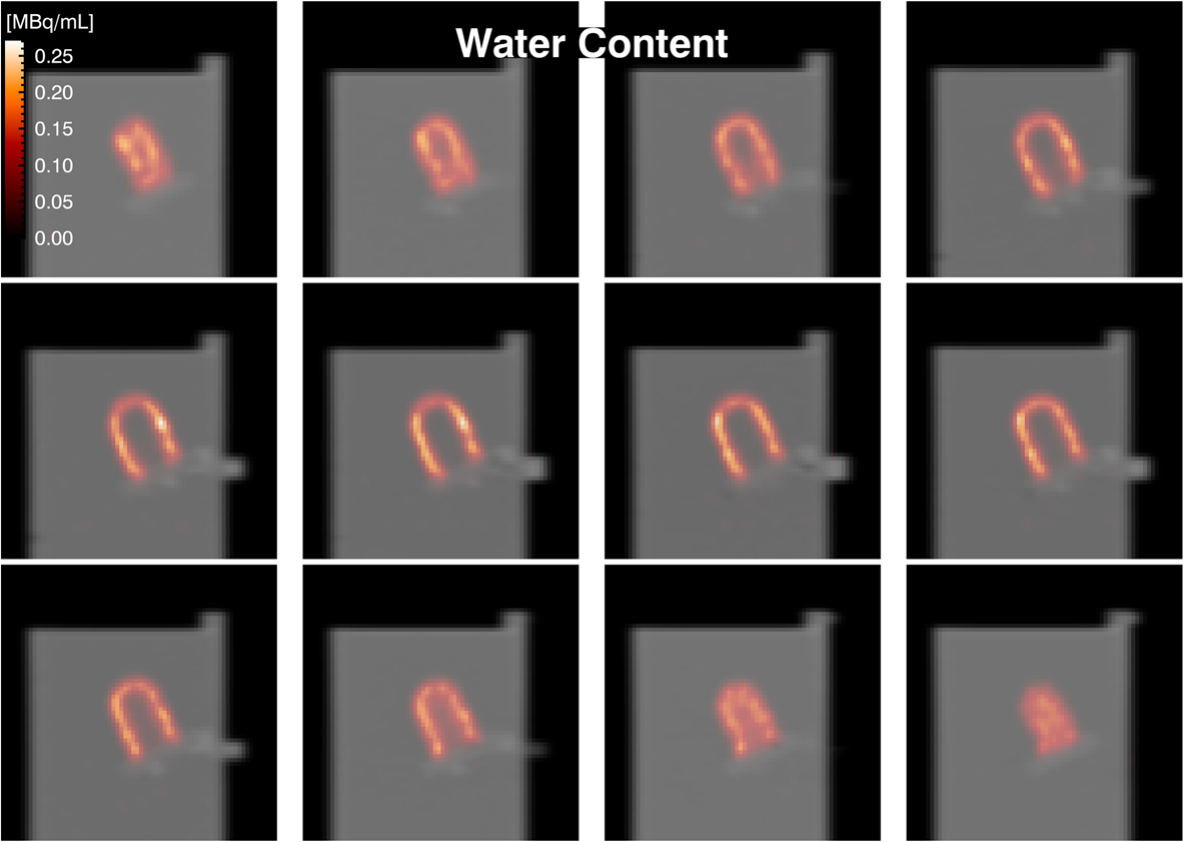
Twelve consecutive SPECT slices (50 iterations) overlaid on the corresponding CT slices for a cardiac insert in a water-filled torso. The ventricle cavity was filled with water. The MCR and CT results are well registered with good contrast between the myocardium and ventricle. Despite the attenuation in the cavity water, the homogeneity of the activity in the myocardium is well recovered, albeit somewhat nosier than in Fig. 7, due to the modelling of attenuation in the forward-projector

Figures 6 and 7 depict 12 consecutive slices of the SPECT volume overlaid on the corresponding CT slices, exhibiting good SPECT-CT registration and myocardium ventricle contrast. The reconstructed myocardiac activity concentration does not show any depth dependence, regardless of torso content. Comparing the two reconstructed images, they appear similar although the image for a water-filled torso is noisier than the one for an empty torso. The total activity in the myocardium was estimated based on a manually delineated VOI [45], and further post-processing was conducted using a morphological dilation operation with a 3 × 3 × 3 kernel [46]. The estimated myocardial activities are presented in Table 3.

**Table 3:** Estimated myocardial activities and relative differences between the estimated and reference activities

Number of iterations	Empty torso		Water content	
	Activity (MBq)	Relative difference (%)	Activity (MBq)	Relative difference (%)
20	21.4	1	25.1	0
40	21.4	1	25.2	0
60	21.3	1	25.2	0
80	21.4	1	25.2	0
100	21.3	0	25.4	0

**Fig. 8:**
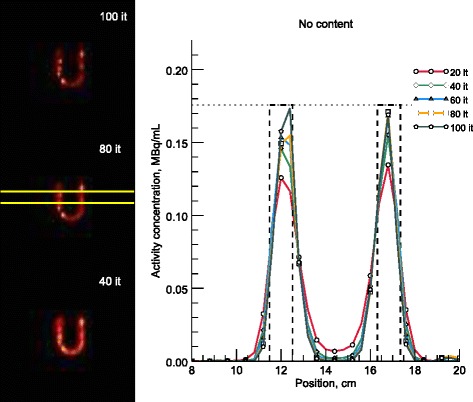
Line profiles over a reconstructed myocardium with the myocardiac insert in an empty torso. On the left are three long-axis images, corresponding to 40, 80, and 100 iterations. The summed profile was calculated from the seven rows in between the yellow markings and includes the data for every 20th iteration. Only alternate data points are marked for clarity. The reference activity concentration, shown by a horizontal dotted line, in the myocardium at the start of the measurement was 0.18 MBq/mL

**Fig. 9:**
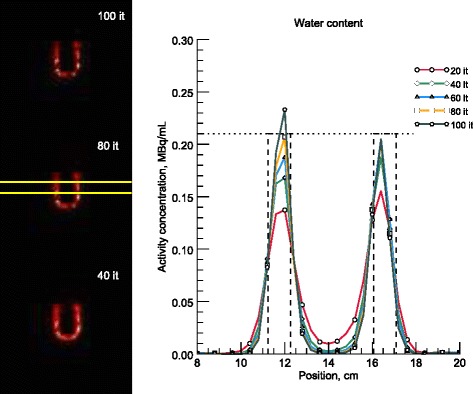
Line profiles over a reconstructed myocardium with the myocardiac insert in a water-filled torso. On the left are three long-axis images, corresponding to 40, 80, and 100 iterations. The summed profile was calculated from the seven rows in between the yellow markings and includes the data for every 20th iteration. Only alternate data points are marked for clarity. The reference activity concentration, shown by a horizontal dotted line, in the myocardium at the start of the measurement was 0.21 MBq/mL

Figures 8 and 9 show line profiles of the reoriented slices corresponding to 20–100 iterations, in steps of 20, for the cardiac phantom with empty and water-filled torsos, respectively. The seven rows used for the average profile are shown in the long-axis images and the approximate boundaries of the myocardium are indicated by the dashed lines.

**Fig. 10:**
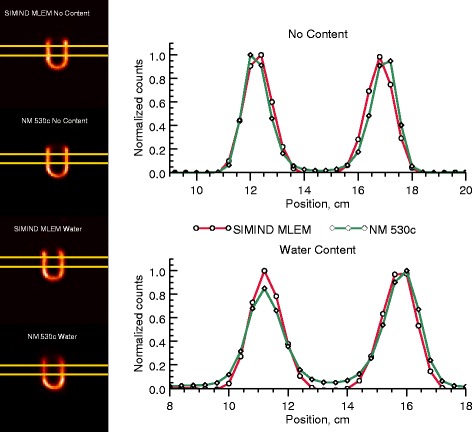
Comparison of line profiles over the myocardium in empty (top) and water-filled (bottom) torsos for MCR and NM 530c reconstruction (40 iterations). The summed profile was calculated from the seven rows in between the yellow markings. A zoomed-in section of the profile is shown in each case to highlight the differences between the two profiles, and each symbol represents a voxel value. The MCR images were filtered post-reconstruction with a Butterworth low-pass filter (cut-off: 0.5 cm^−1^, order: 2)

The MCR and clinical reconstruction results are compared in Fig. 10. The yellow lines in the long-axis images indicate the profile positions. Reconstructions were performed for myocardiac phantoms in both empty and water-filled torsos.

## Discussion

In this paper, we have presented a pinhole reconstruction method that is entirely embedded in the well-known SIMIND MC code. The method has been applied for a dedicated clinical myocardial SPECT system, based on CZT technology, and we have evaluated its usefulness for activity quantification.

Generally, the TARCs were slightly overestimated for all of the spheres, but the estimated sphere activities typically remained within approximately 10% of the reference value (defined by the syringe activity measured in an activity meter and the sphere volumes). A noteworthy exception was the 20% overestimation of the TARC in the 0.5-mL sphere in the empty torso. The trend of total activity overestimation is not evident for the cardiac phantom in Table 3. However, the total estimated myocardial activities in Table 3 depend on the VOI definition used. The VOI used to estimate the myocardiac activity was delineated from a density map and included all of the cardiac phantom voxels and any potential signal spill-out. Increasing the VOI even further caused activity overestimation, especially for the water-filled torso, due to the inclusion of more background voxels (data not shown). The fact that the problem was more pronounced for water suggests that the scatter compensation in the reconstruction was not complete, i.e. that the model underestimated the amount of scatter in the projections. For large VOIs (dilation with a 9 × 9 × 9 voxel mask, i.e. a considerable expansion of the volume), the relative errors were up to 5% for the water-filled torso and up to 2% for the empty torso. The relative errors achieved with MCR are comparable to those previously published. Pourmoghaddas et al. reported relative errors of 5% ± 3% [47] when correcting for attenuation and scatter [20] for a similar measurement setup.

**Fig. 11:**
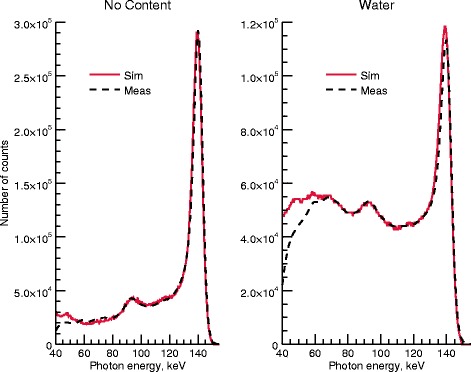
Simulated (solid) and measured (dashed) spectra showing registered counts for different energies. The simulated and measured curves agree well regardless of torso content, proving that both the CZT model and pinhole routine employed in SIMIND are accurate

It should be noted that the MCR activity estimates does not depend on an explicit calibration of the system. Instead, the relationship between the projection counts and activity estimates is a result of the SIMIND camera model only. Hence, the estimated activities are completely independent of a separate activity meter measurements, as well as any errors associated with such measurements. However, this finding indicates that the accuracy of MCR-estimated activity concentrations is highly dependent on the accuracy of the camera model, not only in a relative sense, but also in absolute terms. Examples of sources of uncertainties are the cross sections used in the simulations, both with respect to the mass attenuation coefficients employed for various materials and the estimated density map obtained using CT. Apart from the uncertainties in the camera model, the reference activity is also associated with an uncertainty that would manifest itself as systematic deviations of the TARC and ARC. The CZT model used in SIMIND has previously been validated for sources in air [38]. In order to validate the handling of scatter by SIMIND for CZT, we compared measured and simulated spectra for the sphere geometry described in the “Sphere phantom” section*.* The simulated and measured spectra are shown in Fig. 11. Without any torso content, the two spectra agree well down to 60 keV. The same conclusion is drawn for the case of the water-filled torso despite a slight underestimation of the number of counts in the photopeak. Note that the spectra are absolute (number of counts) and not normalized to their maximum values. From the good agreement, we conclude that SIMIND models photon scattering well.

As shown in Table 1, which presents the MCR results for projections simulated with SIMIND, the total activities for the spheres in the empty and water-filled torsos agree well. So, if the forward-projector used in the reconstruction exactly matched the generation of the measured projections, the errors are substantially reduced. Hence, we believe that the majority of the errors in the measured results originated from model uncertainties in the forward-projector and reference values rather than the approximations used in the back-projector model.

As opposed to the TARCs, the ARCs exhibit volume-dependent underestimation, as demonstrated in Fig. 4. Generally, a smaller sphere means lower recovery. The exception to this rule, the 2-mL sphere in water, can probably be explained by the coarse discretization of the source volume, which made it difficult to delineate the VOIs accurately. Additional errors would occur when the VOI centre point does not coincide with the physical centre of the sphere. The fact that compensation for spatial resolution in the iterative reconstruction did not yield perfect recovery is in line with general resolution compensation experiences [48]. Figure 4 illustrates that the activity concentration recovery continued until the user-selected end-point of 100 iterations for all sphere sizes, albeit slowly for the larger spheres at high iteration numbers. This finding suggests that it would be possible to increase the recovery even further, but at the expense of increasing the computational burden. The same trend of improved recovery and image contrast with increasing number of iterations, even up to 100 iterations, can be seen in the line profiles in Figs. 8 and 9, where the central values approach activity concentrations close to the true activity concentration in the myocardium. Hence, to utilize fully the potential of an accurate forward-projector, such as an MC-based one, more iterations than are typically used clinically may be necessary.

The main drawback of MCR is the long reconstruction time. The overall reconstruction time scaled with the number of iterations and varied with the amount of scattering material, and typical reconstruction times for a heart phantom (50 iterations) were roughly 10 and 20 h for empty and water-filled torsos, respectively. The majority of the reconstruction time is spent in the MC forward-projection simulation step, and any attempts to speed up the back-projection step would have very little effect on the overall reconstruction time. Several variance reduction techniques have been implemented in SIMIND code to improve the simulation efficiency [37]. A specific variance reduction technique, applicable to pinhole simulations, is that photons exiting the source/phantom volume are directed into a solid angle that, for each separate emission point, covers a circular area around the centre of the pinhole having a diameter equal to four times the pinhole diameter. This technique speeds up simulations significantly compared to those in which the emission is isotropic, while still modelling the penetration and scatter effects at the pin-hole edges.

Currently, research is in progress on potential methods of decreasing the calculation time further using parallel-processing message-passing interfaces in the forward-projection, where the simulation can be performed in parallel on multiple central processing units (CPUs). The reduction in simulation time scales roughly with the number of cores used. Since the reconstruction times reported here can be achieved using a single CPU, we believe that this algorithm can be applied in cases in which expedience is not of primary importance, such as for research and post-study evaluations. As the cost of hardware continues to decrease and with the possibility of parallelization, the time aspect is also expected to decrease in importance in the years to come. The reconstruction time could also be reduced by implementing the OS-EM algorithm. In this work, however, we did not utilize OS-EM due to the difficulties in defining subsets from 19 projections.

From an image-quality perspective, the MCR and NM 530c reconstruction results differ somewhat, as shown in Fig. 5. The spheres reconstructed using NM 530c are more uniform due to the noise regularization applied during the NM 530c reconstruction and the Butterworth post-filtering, but are similar in shape to those obtained using MCR. The difference in noise characteristics between MCR and NM 530c can be counteracted by post-filtering the MCR images with a Butterworth filter. For the largest sphere, a ringing artefact is visible in the NM 530c reconstruction but not in the MCR. The relative sphere intensities in the water-filled and empty torsos are similar in the MCR since photon attenuation is included in the forward-projector model, whereas the absence of attenuation compensation in the NM 530c reconstruction led to non-homogeneous sphere intensity suppression. The clinical reconstruction software does allow for attenuation correction, but it is seldom used clinically in our department. Since there are no means of correcting scatter in the clinical program, thereby making a “fair” comparison impossible, we used the clinically employed settings (i.e. no attenuation correction). The myocardium phantom profiles in Fig. 10 for MCR and NM 530c are similar, but the presence of noise regularization produced a more uniform myocardial distribution for NM 530c, as is evident in the individual slices. However, noise regularization could in principle be applied to MCR as well, but its implementation was beyond the scope of this study. Without attenuation correction in the NM 530c reconstruction there is as a negative gradient in the reconstructed activity towards the myocardiac base when the torso is water filled. For MCR, which includes attenuation in the forward-projector, there is no gradient.

One area of potential further development is improvement of the model of the pinhole in the back-projector by accounting for its finite size. This objective could be achieved by analytical determination of the pinhole PSF [43, 47], which would still be an approximation with regard to the model in the forward-projector but would be an improvement over the current ray-tracing model. As an example, the signal spreads due to the finite voxel and pinhole sizes for a point source located 15 cm from the pinhole are 0.14 and 0.68 cm respectively, so for this specific position the total spread is underestimated when ignoring the finite pinhole aperture. Incomplete modelling of the spatially variant PSF affects the noise characteristics in the reconstructed image. In this paper, we determined total activity in VOIs and the cardiac phantom mean activity profiles. In that regard, our results display a good accuracy, but is should be noted that both these measures are fairly resistant to noise. An alternative to calculating the system matrix during reconstruction is to pre-calculate the entire system matrix [33]. Referencing an MC-calculated system matrix in both the forward- and back-projections is optimal from an image quality standpoint, but with the downside of requiring storage of the full or a compressed system matrix.

In general, the full potential of MC-based image reconstruction is not necessarily best demonstrated with a radionuclide-like ^99m^Tc, but rather with radionuclides with more complex emission spectra, such as ^123^I and ^131^I. These radionuclides emit several high-energy photons during their decay that, despite low abundance, can scatter in and penetrate through the collimator edges as well as penetrate though the detector and scatter in the material behind the detector and back into the detector in a way that is not easy to predict using analytical models. Realistic projections can, however, be obtained with full modelling of the radiation transport in the object, collimator, and detector.

## Conclusions

We developed an MC-based reconstruction method for pinhole SPECT and evaluated it using phantom measurements. The combination of an MC-based forward-projector and a simplified analytical ray-tracing back-projector produced quantitative images of acceptable image quality. No explicit calibration is necessary in this method since the forward-projector model maintains a consistent relationship between count and activity.

## Abbreviations

ARC: Activity recovery coefficient
CPU: Central processing unit
CT: Computed tomography
CZT: Cadmium zinc telluride
FOV: Field of view
MC: Monte Carlo
MCR: Monte Carlo-based reconstruction
MIP: Maximum intensity projection
ML-EM: Maximum-likelihood–expectation-maximization
NM 530c: GE Discovery NM 530c cardiac SPECT camera
OS-EM: Ordered-subset–expectation-maximization
PET: Positron emission tomography
SPECT: Single-photon emission computed tomography
TARC: Total activity recovery coefficient
VOI: Volume of interest
